# A Revolutionizing Approach to Autism Spectrum Disorder Using the Microbiome

**DOI:** 10.3390/nu12071983

**Published:** 2020-07-03

**Authors:** Dinyadarshini Johnson, Vengadesh Letchumanan, Sivakumar Thurairajasingam, Learn-Han Lee

**Affiliations:** 1 Novel Bacteria and Drug Discovery Research Group (NBDD), Microbiome and Bioresource Research Strength (MBRS), Jeffrey Cheah School of Medicine and Health Sciences, Monash University Malaysia, Bandar Sunway 47500, Malaysia; dinyadarshini.johnson@monash.edu (D.J.); vengadesh.letchumanan1@monash.edu (V.L.); 2 Clinical School Johor Bahru, Jeffrey Cheah School of Medicine and Health Sciences, Monash University Malaysia, Johor Bahru 80100, Malaysia; sivakumar.thurairajasingam@monash.edu

**Keywords:** clinician, autism spectrum disorder, microbiome, aetiology, comorbidities, diet, prebiotics, probiotics, faecal microbial transplant

## Abstract

The study of human microbiota and health has emerged as one of the ubiquitous research pursuits in recent decades which certainly warrants the attention of both researchers and clinicians. Many health conditions have been linked to the gut microbiota which is the largest reservoir of microbes in the human body. Autism spectrum disorder (ASD) is one of the neurodevelopmental disorders which has been extensively explored in relation to gut microbiome. The utilization of microbial knowledge promises a more integrative perspective in understanding this disorder, albeit being an emerging field in research. More interestingly, oral and vaginal microbiomes, indicating possible maternal influence, have equally drawn the attention of researchers to study their potential roles in the etiopathology of ASD. Therefore, this review attempts to integrate the knowledge of microbiome and its significance in relation to ASD including the hypothetical aetiology of ASD and its commonly associated comorbidities. The microbiota-based interventions including diet, prebiotics, probiotics, antibiotics, and faecal microbial transplant (FMT) have also been explored in relation to ASD. Of these, diet and probiotics are seemingly promising breakthrough interventions in the context of ASD for lesser known side effects, feasibility and easier administration, although more studies are needed to ascertain the actual clinical efficacy of these interventions. The existing knowledge and research gaps call for a more expanded and resolute research efforts in establishing the relationship between autism and microbiomes.

## 1. Introduction

Autism spectrum disorder (ASD) is identified with persistent deficit in social communication and phenotypic behaviours which are typically repetitive and restrictive [1]. This disorder affects more boys than girls in a ratio close to 3:1 [2]. There has been a significant increase in the prevalence of ASD over the decades, it is currently estimated to affect 1% of the general population [3]. The most recent Global Burden of Diseases, Injuries, and Risk Factors Study in 2016 estimates 62.2 million individuals live with ASD globally [4]. It was also demonstrated that the prevalence of ASD based on special education enrolment data within the United States (US) over 11 years, from 2000 (1.2 per 1000) to 2010 (5.2 per 1000) alone shows an increase of 331%. It was inferred that the diagnostic recategorization may be the possible explanation for this significant rise [5]. However, it will be an understatement if it is solely attributable to the changes introduced in the diagnostic criteria of autism and increased awareness which will be explored in this review. It has been speculated that the evolving environmental influence which contributes significantly to the occurrence of ASD could be partly responsible for the rise in the ASD prevalence globally [6]. A rising prevalence in ASD is a matter of concern and calls for a more effective management of this disorder.

The study of human microbiomes has become a prime hope in understanding this disorder and catering to the growth of a more clinical-based intervention more than to merely augment the widely advocated behavioural therapies. The understanding of microbial–human host relationship which was once thought to be purely commensal in nature, if not pathogenic, skewing to a one-sided relationship has now evolved into a complex interaction holding imperative roles in key physiological processes in the human body [7]. Determining the role of the microbiome in human health has become an intriguing quest in recent decades. A plethora of health conditions have been associated with microbiome across a wide range of populations identifying a distinctive spread of microbiome in a selected patient cohort compared to a healthy cohort pointing to its potential etiological role in the occurrence of a commonly identified disorder. It is not an understatement if this could potentially invent a breakthrough management approach in curbing a wide variety of health disorders and pandemics which include obesity and cardiometabolic diseases, infections, respiratory, allergic, gastrointestinal, neuropsychiatric disorders and even cancers [7,8,9,10,11,12,13,14]. The National Institutes of Health (NIH) Human Microbiome Project, launched in 2007, was one of the large-scale projects initiated to support and catalyst the growth of this emerging field of research. The microbiome in five major habitats in the human body, which include the gastrointestinal tract, airway, skin, oral cavity and vagina, were explored in this pursuit [15]. The gut microbiome has been the most widely studied area, accounting for four-fifths of total publications in microbiome over the last four decades [9]. Germ-free rodents and controls—conventionally colonized rodents with specific pathogen free (SPF) rodent models—have been utilized to ascertain the microbial influence in behavioural outcomes which have been grouped into four domains including, ASD-mimicking behaviours, stress and anxiety-related behaviours, learning and memory and motor controls. The germ-free rodents exhibited significant deficits in social interactions, cognitive and motor functions compared to SPF rodents suggesting the imperative role of microbiome in neurobehavioral outcome [16]. The gut microbial composition, if disturbed, has an impact on the various physiological activities regulated by these microbes principally through its metabolites and has a bidirectional communication with the brain involving autonomic nervous system. Neuronal, neuroendocrine and immunologic pathways have been described through which the microbes contribute to the bidirectional signalling between the gut and the brain [17,18]. The bidirectional transfer of information between the gut and the brain is principally controlled by the vagus nerve. The gut microbiota communicate to the brain via endocrine and neurocrine pathways while the brain impacts the microbial composition via immune and humoral systems mediated by autonomic nervous system, thus establishing the gut–brain–microbiota axis [18,19]. In the context of ASD, the exploration of other habitats of the microbiome, in the vagina and oral region, highlights the influence of maternal factors in the development of ASD. The vertical transmission of disrupted maternal vaginal microbes to the offspring at birth predisposes the offspring to the prenatal risk of developing ASD [20]. The direct relation of the oral microbiome and ASD has yet to be established; however, the resemblance of infants’ oral microbiome to the maternal microbiome during the first six months of birth ascertains the significant contribution of maternal microbiome in early stage of oral microbial colonization [21].

Although the aetiology of ASD largely remains unanswered, the emerging microbial knowledge may be a key finding in explaining the etiopathogenesis of ASD; however, with more extensive work needed to understand its involvement at the molecular level. In an attempt to understand the molecular involvement in ASD, a study on post-mortem brain tissue and small intestines of ASD subjects revealed that blood–brain barrier (BBB) and gut barrier were disrupted with significant neuroinflammation evident by increased expression of genes and markers associated with brain inflammation. It was further inferred that the gut–brain axis disruption may be associated with non-self-antigens which triggers neuroinflammatory reaction by crossing the damaged gut barriers, thus leading to ASD in genetically susceptible subjects [22]. The BBB has a pivotal role in early phase of brain development and neuronal functions [23,24,25]. It was found that adult germ-free mice models and the foetuses of germ-free mice’s mothers had more permeable BBB compared to mice with pathogen-free gut microbiota. The faecal transfer from a pathogen- to germ-free mice models and administration of short-chain fatty acid (SCFA) producing bacteria were able to restore the permeability of BBB, emphasizing the roles of intestinal microbes and SCFA in guarding the integrity of BBB [26]. The genetic component has primarily conditioned ASD as a highly heritable disorder through twin studies and large population-based studies which assess familial risk [27]. Nevertheless, the influential role of environment may potentially supersede the sole genetic involvement in the development of ASD, especially with the recent data pointing to the integrative approach of epigenetics in the study of pathogenesis of ASD [28]. In a study involving 192 twins, genetic factors were made accountable for only 38% of ASD risk, whereas the remaining 68% was attributed to environmental factors [29]. The microbial composition is regulated and influenced by many factors which could be broadly classified into extrinsic and intrinsic factors. The extrinsic factors consist of mainly environmental elements which include diet, lifestyle, microbial exposure in early developmental phase and infection, whereas intrinsic factors are naturally occurring elements within an individual which include genetic make-up, metabolites, immunologic and hormonal aspects [30]. The understanding of microbial involvement as part of an interplay between the intrinsic and extrinsic factors gives rise to an immense possibility of associating various factors in explaining its relation to ASD; however, the research progress in understanding the actual etiological mechanism involved in ASD development is still at an infant stage with a lack of defined explanations [18,31,32]. The co-existing health conditions in ASD is another major challenge in both research and clinical fronts. At least one comorbidity exists in about 70% of children with ASD, while 41% have two or more, reflecting the onerous disease burden in this population [33]. There are limited numbers of published studies on comorbidities alone in ASD due the complex and large spectrum of heterogeneity associated with this disorder. The widely reported and studied medical and psychiatric comorbidities in ASD include gastrointestinal (GI) disorders, epilepsy, depression and anxiety disorder [33,34,35]. These comorbidities can be individually linked to the microbiome which either overlaps with a similar ASD pattern of microbial dysbiosis, or that the related use of medication induces microbial dysbiosis which could contribute to ASD occurrence. This relation points towards the need to include the assessment of associated comorbidities to further understand the possible shared aetiology in ASD development, as many microbial studies using ASD subjects often do not take into account their existing comorbidities.

This review therefore focuses on autism spectrum disorder and microbiome in general while examining its relation to the hypothetical aetiology of ASD and its commonly associated comorbidities.

## 2. Evolving Conceptualization of Autism Spectrum Disorder

ASD is a behaviourally defined, neurodevelopmental disorder which lacks specific clinical biomarkers and has seen an evolving conceptualization over the last seven decades since it was first described [36,37]. The Centers for Disease Control and Prevention (CDC) reports that the earliest known median age of ASD diagnosis is at 52 months in the United States (US), whereas in the United Kingdom (UK), the median age of ASD diagnosis is reported at 55 months [38,39]. In general, ASD is diagnosed by three years of age in most children, although roughly 39% of children are not first evaluated until after four years of age [38]. A psychiatric diagnosis which has behaviour as its basis of definition heavily relies upon a meticulous observation and clinical expertise as it lacks standardised biomarkers [40]. Therefore, it is crucial to understand the core concept of a disorder to facilitate a more effective diagnostic process. The idea of autism emerged as early as in the 19th century when a Swiss psychiatrist, Eugen Blueler described the aloofness he observed in individuals with schizophrenia as a form of schizophrenic trait itself [41,42]. It was in the 1943, when two child psychiatrists, Leo Kanner and Hans Asperger, garnered the attention of scientific community through their publications, which eventually led to recognising autism as a distinct category of diagnosis in children years later [42]. They individually published clinical cases of children with distinctive behaviours, primarily reflecting social deficits and echoing the descriptive term, autism coined by Blueler [43,44]. However, autism was often identified as a form of schizophrenia until a more refined and stand-alone diagnostic classification was first introduced in 1980 by American Psychiatric Association in Statistical Manual of Mental Disorders (DSM-III) and subsequently by the World Health Organization in International Statistical Classification of Diseases and Related Health Problems (ICD-10) in 1990 [36]. These are the two broadly adopted sources of reference for both research and clinical purposes with revised versions to date. The most recent definition, based on the DSM-5 published in 2013, includes subtypes of autistic disorders, Asperger’s syndrome and Pervasive developmental disorder not otherwise specified (PDD-NOS) under one umbrella term known as ASD. DSM-5 allows classification of ASD based on its severity and takes into account intellectual ability and other comorbidities [1]. Despite an evolving conceptualization from a disorder to a spectrum highly denoting its clinical diversity, the core definition of autism has remained central to deficits in social interaction and stereotypic behaviours. It took several decades to define ASD within a smaller framework of classification to facilitate diagnostic process. However, it still remains a debate to perfectly fit ASD in a confined framework of definition due to the heterogenous expression of the core symptoms which are further influenced by age and development factors. The progressive work to define ASD within a narrower framework of theoretical concept inadvertently reflects the complexity that revolves around this neurodevelopmental disorder, which makes it even more challenging to conclusively define other aspects pertaining to ASD.

## 3. Microbiome and Autism Spectrum Disorder

**Figure 1:**
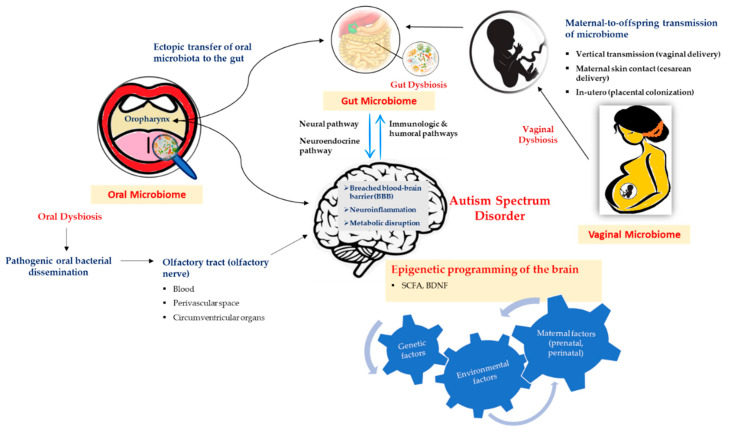
Illustration of autism spectrum disorder (ASD) and its association with microbiome.

The terms microbiota and microbiome have a slight semantic difference, however, collectively refer to the entire microbial community residing on and in human body encompassing bacteria, eukaryotic viruses, fungi, protozoa, archaea and bacteriophage. The number of bacteria is overwhelmingly larger than the other taxa to an extent where microbes interchangeably could simply refer to bacteria alone. Our human body is home to trillions of microbial cells which encode 100-fold more genes than human genome, with the latest revision estimating a ratio of 1.3 bacterial cells for every human cell, showing a reduction from widely quoted 10:1 and 100:1 ratios, respectively [15,45,46]. The enormous spread of the microbiome in the human body has both therapeutic and pathogenic roles in health depending on the microbial composition [47]. In the context of ASD (Figure 1), the link between gut, vaginal and oral microbiomes and ASD have been studied thus far using animal models and human subjects.

Figure 1 illustrates the possible mechanism involved in the microbiome–brain interaction in the context of autism spectrum disorder (ASD). The neural, neuroendocrine, and immunologic and humoral pathways are the potential mediators in the bidirectional communication between the gut microbiome and the brain. The maternal contribution is significant in determining the early intestinal colonization in the offspring while in general, the environmental factors that significantly alter maternal microbiome during the prenatal and perinatal periods influence the microbial composition in the offspring. Ectopic transfer and dissemination of pathogenic oral bacteria mediated by the olfactory nerve via the blood, disrupted blood–brain barrier (BBB), perivascular space and circumventricular organs to the gut and brain, respectively, are plausible mechanisms resulting in neuroinflammation and metabolic disruption in the brain, thus indicating the influence of oral microbiota and dysbiosis in ASD occurrence. Another plausible exchange pathway in the gut–brain axis is thought to be mediated by the oropharynx which has a significant role in the pathology of ASD. In general, the interaction between microbiome and the brain in the context of ASD involves a complex mechanism and interplay between the genetic and various environmental factors, in which, some could be explained through epigenetic mechanisms involving short-chain fatty acids (SCFAs) and brain-derived neurotrophic factors (BDNFs).

### 3.1. Gut Microbiome

Gut microbiome has been the most extensively explored area in relation to ASD compared to microbiome in other habitats of the body. The years between 2013 and 2017 saw the largest number of publications focusing on gut microbiota alone accounting for more than 80% of total publications on microbiota in the last four decades, implying the immense possibility of gut microbe in relation to human health [9]. The human gut is the largest reservoir of bacteria in human body which has imperative physiological roles in metabolism, digestion, immunity, endocrine and neurological activities [48]. The gut microbes interact closely with multiple human cells and any imbalance in the microbes, otherwise known as dysbiosis, will have an impact on host key physiological processes and has a potential aetiological role in many health conditions [47]. The immense possibility of gut microbes in human health has prompted researches to highlight the importance of recognizing it as an individual organ system in the human body [49].

In ASD, the relation between gut microbes and central nervous system resulting in manifestation of ASD behaviour has been established using rodent models and clinical subjects, although the latter is still largely inadequate to strongly affirm the causative relation between gut microbes and ASD symptomatology. *Bacteroidetes* (e.g., *Bacteriodes* and *Prevotella*), *Firmicutes* (e.g., *Lactobacillus*, *Clostridium*, *Ruminococcus*), *Proteobacteria* (e.g., *Enterobacter*) and *Actinobacteria* (e.g., *Bifidobacterium*) are four major phyla that are constituent of a healthy adult gut with *Bacteriodes* and *Firmicutes,* representing more than 90% of gut microbes [45,50]. It was found that, in the ASD population, the ratio of *Bacteroidetes* to *Firmicutes* phyla were increased compared to neurotypical cohort [51]. This pattern is also observed prominently in Western ASD children and has been inferred to be influenced by environment and dietary habits [52]. There is also another study which found increased *Firmicutes* compared to *Bacteroidetes* in the ASD group compared to neurotypical group where both groups have GI symptoms [53]. A large number of gram-negative bacteria (e.g., *Bacteroidetes*), exhibit pathogenic nature because of their cell wall which contains lipopolysaccharide (LPS) that has malefic effect on the immune system of the host. LPS confers the ability to breach the blood–brain barrier, increasing the mercury level in the cerebrum and decrease glutathione level which is a key antioxidant in the detoxification of heavy metals. Two species of bacteria with LPS in their cell walls, namely *Bacteriodes vulgatus* and *Desulfovibrio*, have been significantly raised in ASD children compared to neurotypical children. The pathogenic feature of these bacteria may possibly contribute to ASD development. Another noteworthy genus of gram-negative bacteria is *Prevotella*, a healthy-gut biomarker which is at a greater abundance in neurotypical group while almost absent in the autistic group. *Prevotella* is found in abundance in individuals whose diet is rich in plant-based carbohydrates and includes fish oil which is vital to normal brain development. It has a metabolic role in vitamin B1 production which is known to alleviate ASD symptoms. The lack of *Prevotella* suggests a distinctive dietary habit in autistic children which significantly alters the gut microbial composition and has influence on neurodevelopment which points to its therapeutic potential if restored [48,54]. Another concerning microbe is *Clostridium*, a gram-positive genus in the *Firmicutes* phyla, which has been found higher in ASD group. *Clostridum boltae, C. histolyticum*, and subgroups of *I* and *XI* are the associated species. These gram-positive microbes release enterotoxins which damage the intestinal tissue resulting in diarrhoea and may cater to the increased absorption of large molecules like casein and gluten. On the other hand, beneficial bacteria *Bifidobacterium* were found less abundant in the ASD group [48]. The increased abundance of potentially pathogenic bacteria and decreased beneficial bacteria affirms the existence of gut dysbiosis in the ASD population. In another perspective, the general lack of gut microbial richness and diversity in ASD group, predisposes them to a vulnerable gut environment which could lead to GI disturbances, infections and autistic behaviours [48]. In general, a significant alteration in gut microbial composition interferes with key physiological processes which have an influence on the neurobehavioral manifestation of ASD symptoms either through the absence of beneficial microbial metabolites, release of harmful microbial endotoxins, pathogenic invasion of the intestinal wall and/or through immune mediators catering to neuroimmune inflammation [6,47,48,50,55].

Many factors have been associated with the dysbiosis of gut microbes which include diet, medication and hygiene as well as numerous maternal factors which include maternal stress, infection and a high-fat diet during pregnancy [6,56,57,58,59]. The frequent use of oral antibiotics in ASD children during the first three years of life is another factor that has also been hypothesized to disturb the natural balance of the gut microbes while some antibiotics confer benefits [48,55,60]. For instance, the use of macrolides except penicillin within the first six months has been associated with decreased *Actinobacteria* and increased *Bacteroidetes* and *Proteobacteria* and this microbial alteration persisted for a year [61]. On the other hand, the administration of oral vancomycin in a small eight-week clinical trial, which targets gram-positive bacteria including *Clostridium*, temporarily but significantly relieves gastrointestinal and ASD symptoms in 8 out of 11 children with regressive onset autism [62].

### 3.2. Vaginal Microbiome

The vaginal microbiome denotes the influence of maternal factors in the occurrence of ASD. As it has been understood that we are born germ-free, the first colonization of microbes in human gut is thought to begin at birth while passing through the vagina, although there are emerging data suggesting it occurs even earlier in utero via placental colonization [63,64]. In cases of caesarean delivery, the colonization occurs after in contact with the maternal skin and environmental microbes [65]. The passing of microbial community through vertical transmission determines the child’s gut microbial composition, thus any significant disturbance to maternal vaginal microbe (e.g., reduced *Lactobacillus*) inadvertently interferes with the normal neurodevelopment in offspring due to high metabolic demand during the critical time of early brain development [20,66].

The vaginal tract houses more than fifty microbial species, dominantly populated by *Lactobacillus* in a healthy woman [67]. Maternal stress during early pregnancy has been found to exert a suppressive effect on the vaginal immune response and the abundance of *Lactobacillus*, thus resulting in gut microbial dysbiosis in offspring via vertical transmission [66]. Bacterial vaginosis, a common infection among women characterized by a marked reduction in *Lactobacillus*, predisposes a pregnant woman to the risk of preterm delivery [68]. It has been reported that infants born extremely preterm have 10-fold higher risk of developing ASD compared to infants born at term [69,70]. Further, numerous clinical studies and large epidemiological studies have ascertained that prenatal maternal infection and elevated level of pro-inflammatory cytokines increases the risk of ASD in offspring. The injection of antigens activating such maternal immune response in pregnant mice, rats or monkeys have resulted in neurobehavioral deficits mimicking ASD in their offspring [71,72].

### 3.3. Oral Microbiome

Oral cavity is home to more than 700 bacterial species or phyla of which more than 50% are yet to be cultivated and these microbes have an influence on individual oral health. Poor oral health is one of the concerning issues amongst ASD children and it is more prevalent in this group compared to neurotypical group [73]. In the context of ASD, only a handful of studies have explored the differences between oral microbiota in autism and neurotypical children. Notable differences in the distribution of oral microbes were detected in ASD children compared to neurotypical children [54,74,75]. In one of the largest cross-sectional studies which studied the oral microbiome profiles in ASD and typically developed children, eight oral taxa that could distinguish children with ASD from typically developed children were identified. Further, 28 taxa that distinguish ASD children with and without GI disturbances were also identified. It was inferred that the gut microbial disruption could potentially extend to the oropharynx. The analysis of oral microbiome to aid the clinical diagnosis of ASD was also suggested [74].

ASD children often have speech-related difficulties and are very selective with food choices where each of these have motor and sensory involvement, respectively. Oropharynx serves as an important bridge to the GI tract and is innervated by five cranial nerves of both motor and sensory origins. It is thought to play a significant role in the pathology of ASD and has a plausible exchange pathway in the gut–brain axis [74,76,77,78]. Other plausible pathways in which the oral bacteria could reach the brain have also been hypothesized resulting neuroinflammation and metabolic disruption in the central nervous system. It has been hypothesized that the olfactory nerve in the olfactory tract may act as a potential mediator in bacterial dissemination to the brain through blood, disrupted blood–brain barrier, perivascular spaces or circumventricular organs [79,80,81]. Further, similar distribution pattern of gut and oral microbiota were demonstrated in a study of oral microbiota in ASD children with significantly higher amount of *Proteobacteria* and *Firmicutes* and lower amount of *Bacteroidetes* and *Actinobacteria* suggesting a potential interaction between gut and oral microbiota leading to a shared pathway in the etiopathology of ASD [54,75,81]. It was also found that oral bacteria resemble 45% of the stool bacteria in the Human Microbiome Project, suggesting a possible interaction between the gut and the oral microbiome [82]. Another example is the increased oral *Bacilli* genus (*Firmicutes* phylum) in ASD children which is also found in abundance in the gut of ASD children and those with inflammatory bowel disease [75,83]. This intestinal colonization by oral bacteria has been hypothesized to occur through ectopic transfer of pathogenic oral bacteria (e.g., *Porphyromonas gingivalis* in chronic periodontitis) to the gut which could induce gut microbial dysbiosis and trigger systemic inflammation [81,84,85]. Poor oral health and hygiene, dental caries and lack of dental care have been found more prevalent in ASD children, implying that ectopic transfer of pathogenic bacteria to the gut associated with oral dysbiosis may possibly explain its relation in ASD occurrence [86]. A marked increase in potential pathogens in analysis of oral samples of ASD children which include *Streptococcus* and *Haemophilus* and reduced abundance of beneficial bacteria like *Prevotella* also point to a significant oral microbial dysbiosis in the ASD group. It is hypothesized that *Haemophilus parainfluenza*, a gram-negative bacteria associated with oral diseases, and its metabolites could potentially cross the blood–brain barrier and impose a detrimental effect on brain development which results in ASD [54,87].

There are specific bacterial species found in subjects with periodontal disease which were otherwise not detected in individuals with a healthy oral cavity [88]. Periodontal disease is associated with an increased risk of preterm birth by 2 to 7 times [89]. Microbial species which were detected in the oral cavity and not in the urogenital tract have been found as causative organisms in intrauterine infection which confers a high risk for preterm birth [90]. More interestingly, the microbiome of 48 term placentae were found to resemble oral microbiome more compared to vaginal microbiome with theory suggesting hematogenous dissemination, especially with underlying periodontal disease, and oral sex may be possible route for such colonization resulting in intrauterine infection [91,92,93]. The prenatal maternal infection which increases the risk of preterm birth inevitably pave the way for ASD occurrence risk in infants born to mothers with the associated conditions which implies a possible but indirect association with the maternal oral microbiome. The maternal influence was more significant during the infant stage and early childhood. During the first six months after birth, 85% of the oral microbiota of infants resembled their mother’s [21]. The pregnancy term, mode of delivery and feeding method were other identified factors that influence the development of oral microbiota in early childhood [94].

## 4. Aetiology of Autism Spectrum Disorder

The aetiology of ASD has always remained a puzzle that has yet to be fully understood. The diverse expression of ASD symptomatology, its associated risk factors and comorbidities make it difficult to pinpoint the exact mechanism involved in the etiopathology of ASD. Nevertheless, the aetiology of ASD can be broadly classified into genetic and environmental causes. Although these are not direct causative factors, strong associations have been established and linked to the development of ASD. Individually, genetic and a myriad of environmental factors have been identified to contribute to the occurrence of ASD through various mechanisms. The microbes and microbiome have both environmental and genetical origins in the manifestation of ASD. More interestingly, a complex interaction between gene and environment through an epigenetic mechanism has also been implicated in the pathogenesis of ASD [95,96,97].

### 4.1. Genetic Factors

Heritability confers a large accountability in cases of ASD, with an estimate of 83% of familial risk in a meta-analysis of twin and family studies. This reanalysis sees a drop in the percentage risk compared to previously published meta-analysis of twin studies which estimates heritability ranging between 64% and 90% with minimal influence of shared environmental factors between the twins [27,98]. The diverse clinical phenotype in ASD is due to the genetic heterogeneity in the ASD population, particularly when comorbid conditions exist. Both common and rare genetic variations which are either heritable or occurs newly as de novo mutation (DNM) contribute to the occurrence of ASD [99,100]. Rare genetic variants have a larger effect compared to the smaller effect of common variants in ASD phenotypes and can combine to create an ASD risk [101]. DNM is a rare variant and newly occurs during gamete formation or at the early phase of embryonic development which are not inherited from either of the parents and unique to the child, resulting in the sporadic occurrence of ASD. DNM is more frequently associated with ASD subjects with co-occurring intellectual disability or developmental delay [102,103]. DNM and common genetic variants provoke the idea of environmental influences which could potentially surpass the etiological weightage of genetic factors and heritability in ASD.

Despite the high accountability to genetic origins, there is no individual genetic marker that has been identified to date. However, continuous attempts are being made to identify novel genes which are significantly attributable to ASD. In a meta-analysis which was attempted to identify the genetic risk of ASD, two novel ASD risk genes, namely, YBX3 and HSPA1A, were found to be associated with the pathogenesis of ASD through an indirect regulation of neuronal pathways involved in behavioural manifestation of ASD [104]. These genes confer protective mechanism against the development of ASD, but it was later found to be of a weak association [105]. This further ascertains the lack of homogeneity inevitably challenges the genetic evaluation in ASD populations.

### 4.2. Environmental Factors

A vast number of environmental factors have been linked to the development of ASD. The role of environment has been speculated to begin since pre-conception and extends to post-natal period in a child. Pre-conceptionally, advanced paternal age and maternal age at more than 50 and 40 years old, respectively, confers a significant risk for ASD development, apart from low level of education status in parents [106,107]. The greatest number of environmental factors have been linked during the prenatal period, particularly during the first and second trimester of pregnancy which include maternal infections, comorbid cardiometabolic conditions, certain antidepressant and antiepileptic medications, toxic exposures, diet and lifestyle. Perinatal factors which include mode of delivery, obstetrics complications, prematurity, hypoxia, low birth weight and low Apgar score have been associated with ASD risk [108,109]. Postnatally, congenital infection, the use of steroid therapy in very low birth weight infants, birth asphyxia and neonatal jaundice have all been found more prevalent in children with ASD [110,111]. The environmental factors may be explained by direct biological impact on neuronal activities of developing brain of the foetus or foetal activation of neuroinflammatory responses and gene dysregulation which are hypothesized to result from maternal immune activation which crosstalk through the placenta [109,112]. Largely, these environmental factors have been studied retrospectively in cohorts of ASD children and mothers of ASD children, while only limited number of studies done using rodent models. There are studies which deem environmental factors to play a more prominent role in the liability and genetical variance of ASD, thus paving way for the eminent role of epigenetics in ASD [96,113,114].

### 4.3. Epigenetic Factors

While traditional methods of identifying genes and environmental factors independent of each other has seen a lack of integrative approach in studying the aetiology of ASD, epigenetics has become the prime pursuits in recent decades incorporating the role of environmental influence on genes. Epigenetic refers to non-genetical influences which changes the phenotypic expression of a gene without actual mutation or alteration occurring in the original DNA sequence and these changes are heritable [97]. It is an epitomic description of a gene–environment interaction in ASD which can be explained by integrating various environmental factors at a cellular level in the occurrence of ASD, particularly through gut microbiota-derived metabolites. At the molecular level, the epigenetic modification occurs via two broadly studied mechanisms, which include DNA-methylation and histone modification besides Ribonucleic acid (RNA) interference [28,115,116,117]. Epigenetic programming of the brain explained through stress-regulating pathways and reduced expression of brain-derived neurotrophic factor (BDNF) by environmental factors during pre-natal and postnatal periods have been thought to exert a long-lasting effect on the neural functioning and behavioural outcome [118,119]. BDNF has a crucial role in the regulation of neurodevelopment, neuronal functions and neuroplasticity and it is frequently associated with depressive disorder and neuroinflammation [120,121]. However, recent findings on new-borns later diagnosed with ASD have demonstrated a significantly reduced level of BDNF in the blood samples [122]. In mice models, decreased levels of BDNF transcript variants were observed in germ-free mice models and antibiotic-treated SPF mice models in the amygdala regions [123,124,125]. These associations of BDNF with the epigenetic mechanism, ASD subjects as well as microbiome, warrant the need to further study and explore the role of BDNF in relation to ASD as currently there are no available studies to affirm this relationship.

The gut microbiome is thought to modulate gene expression through an epigenetic mechanism which may either institute the etiopathogenesis of ASD and/or aggravate ASD symptomatology primarily [126]. It has also been hypothesized that the epigenetic regulation is diet-dependent where the role of microbial metabolite, SCFAs come into play. SCFAs are the product of the ASD-associated bacterial (such as *Clostridia, Bacteriodetes, Desulfovibrio*) fermentation of dietary carbohydrates and are regulated by the gut microbial composition [115,127,128]. However, an overproduction of SCFAs (e.g., propionic acid, butyrate, acetate) have been indicated in ASD based on analysis of faecal samples of ASD children and it was inferred that this possibility could be due impaired fermentation process and utilization of its by-products without eliminating the possibility of increased faecal SCFA could also be due to an overall increased SCFA production in ASD children, which is indeed regarded beneficial [129,130]. Therefore, the microbial dysbiosis (reduced SCFA-producing, ASD-associated bacteria) manipulates the level of nutrients and metabolites like SCFA which regulate the DNA methylation and histone modification resulting in immune activation that have a potential role in ASD development. These metabolites either directly inhibit the enzymes which catalyse these epigenetic processes or alter the substrate availability required for the enzymatic process, thus highlighting the possible epigenetic mechanism in the occurrence of ASD [127].

## 5. Comorbidities in Autism Spectrum Disorder

Gastrointestinal comorbidities have been found to be significantly higher and more common in the ASD group compared to neurotypical children, with 46.8% of ASD children exhibited at least one GI symptom [131,132,133]. GI disorders have been associated with gut dysbiosis, although whether underlying GI disturbances result in dysbiosis or the dysbiosis results in GI disturbance largely remains a chicken-or-the-egg conundrum. Gastrointestinal symptoms (e.g., constipation, diarrhoea, bloating) have been correlated to gut dysbiosis and severity of autistic symptoms [6,65,134,135]. It was hypothesized that the gut dysbiosis gives rise to the pathogenic invasion of intestinal wall, thus resulting in the breach of the gut barrier which ultimately results in neuroinflammation responsible for ASD’s behavioural manifestation [9,48]. However, some of the recent findings demonstrated reduced diversity and altered microbial pattern in autistic group compared to a neurotypical group; however, no significant correlation was found between GI symptoms and severity of ASD symptoms [31,48,136,137]. It was further inferred that GI disturbances alone could possibly trigger ASD symptoms possibly due to heightened sensory perception and experience in ASD children [134]. An interesting correlation between GI symptoms, intestinal mucosal dysbiosis, gene expression and ASD was highlighted in a study which looked at the molecular mechanism involved in explaining the GI disturbances in ASD children by analysing the intestinal biopsies obtained from 15 children with ASD and GI disease, and seven children with GI disease alone. The messenger ribonucleic acid (mRNA) deficiencies in genes encoding disaccharidases and hexose transporters responsible for carbohydrate digestion and transport across the intestinal epithelium was linked to the intestinal dysbiosis (decreased *Bacteroidetes*, increased *Firmicutes* to *Bacteroidetes* ratio, and increased *Betaproteobacteria*) in the ASD group. The pre-existing impaired carbohydrate digestion and absorption manifests as GI disturbances with dietary intake of carbohydrates which leads to fermentation, increased gas production and osmotic load in the gut of ASD children. Although no dietary evaluation was done in the children involved in this study, its finding provides a significant insight regarding the GI disturbances in ASD children, especially on how gluten-free/low-carbohydrate diets may benefit some of the ASD children [53]. In another study, the restoration of microbial imbalance with significant and lasting improvement in terms of GI symptoms and autistic behaviours in children diagnosed with ASD was made possible through microbiota transfer therapy (MTT) [138]. This highlights the influence of microbiome in both GI symptoms and autistic behaviours; however, no distinctive microbial patterns specific to ASD subjects, with or without the presence of GI disturbances, have been elicited so far.

Epilepsy and ASD have often been associated with one another; however, the possible pathophysiological link has yet to be fully understood. It has been reported that ASD subjects possess a seven-fold increased risk of developing epilepsy compared to general population, with an estimated prevalence of epilepsy in ASD up to 50% [139,140]. The prenatal use of anti-epileptics, mainly valproate, has a teratogenic effect on the neurodevelopment of the offspring and predisposes them with a risk of developing ASD [141,142]. The valproate has been demonstrated to cause microbial disruption in mouse models of ASD in the context of in utero usage [143]. Interestingly, ketogenic diet in ASD cases with refractory epilepsy has shown significant improvement in both ASD symptoms and seizure episodes through gut microbial modulation [144]. *Akkermansia* and *Parabacteroides* are two ketogenic diet associated microbes which confer the anti-seizure effect [145].

Depression and anxiety are part of neuropsychiatric disorders which have been elucidated through the gut–brain–microbiota axis [146]. These disorders are associated with the alteration of gut microbial composition, thus impacting the neurobehavioral outcome as narrated in the context of ASD [147,148]. Similar to gut microbial observation in ASD subjects, the ratio of Bacteroidetes to Firmicutes phyla were markedly increased in both animal models and human subjects with depression [149,150]. Further, a recent review on psychotropics and the microbiome has highlighted gut dysbiosis and anti-microbial exertions of antipsychotic, antidepressant and antianxiety drugs which are commonly prescribed in ASD subjects with associated psychiatric comorbidities [151,152].

## 6. Microbiota-Based Interventions and ASD

### 6.1. Dietary and Supplementary Interventions

Diet has an influential role in determining the intestinal microbial composition and in rodent models; it has been demonstrated that significant dietary change has the potential to alter the microbiome as rapidly as over one day [153]. The microbiota evaluation of ASD children differed across countries, suggesting the influence of geographical location in microbiota profile, and diet has been thought to be an important factor to explain such differences [32,154]. SCFA, the colonic by-product of bacterial fermentation of dietary fibres and resistant starch, is gaining attention as one of the important mediators of the gut microbiota–brain communication, although the exact mechanism on how this metabolite influences the brain physiology and neurobehavioral outcome is yet to be fully understood [155,156,157,158,159]. An intervention using a diet that promotes increased SCFA levels (Mediterranean diet, plant-based proteins, dietary fibre) may potentially exert a positive impact on ASD behavioural outcomes, although no studies have been done so far to support this relation in human subjects [158]. Dietary intervention which has been commonly advocated amongst ASD children include gluten- and casein-free diets which have been reported to confer a beneficial outcome in ASD symptoms, while more recent study shows no significant changes in ASD symptoms after a six-month trial [160,161,162]. This intervention is recommended only when there is a clinically diagnosed intolerance to gluten and casein [162]. In terms of maternal aspect, rodent models have significantly demonstrated that offspring born to mothers fed with high-fat diet eight weeks before mating had impaired social interaction and repetitive behaviours mimicking ASD, with altered gut microbial composition and notable nine-fold decrease in *Lactobacillus reuteri* [163]. The Western diet, also referred to as the high-fat diet, has been associated with reduced microbial diversity and richness [164]. Dietary intervention should be exercised with caution to avoid malnourishment and nutritional deficiency. Vitamin D deficiency has been commonly reported in ASD children and low prenatal vitamin D has been associated with an increased risk of ASD occurrence [165,166,167]. It was also found that GI problems are more evident in ASD subjects with vitamin D deficiency than those without this deficiency [166]. The supplementation of vitamin D_3_ shows a remarkable improvement in ASD core symptoms [168].

### 6.2. Prebiotics, Probiotics, Synbiotics and Antibiotics

Prebiotics are mainly fibres consisting of non-digestible diet components which benefit the host’s health by selectively promoting microbial proliferation in the colon, generally *Lactobacilli* and *Bifidobacteria*. The probiotics are live, non-pathogenic microorganisms that confer health benefits to the host when adequately administered, whereas synbiotics are formulations consisting of both prebiotic and probiotic which were meant to improve the efficiency of probiotics [169]. These interventions have been recommended as adjuvant therapies given their promising benefits in terms of improving ASD behavioural symptoms and ameliorating GI disturbances in ASD children [170,171]. The administration of probiotics consisting of three strains (*Lactobacillus acidophilus*, *Lactobacillus rhamnosus* and *Bifidobacteria longum*) for a duration of three months significantly improved autistic behaviours and GI symptoms in ASD children with increased count of the beneficial bacteria *Bifidobacteria* and *Lactobacilli* [170]. In a pre-clinical study, *Bacteroides fragilis* has also been demonstrated to restore the microbial dysbiosis, improve gut permeability and autism behaviours in the offspring of maternal immune activation (MIA) mouse models which displayed ASD behavioural features [172]. In ASD children, probiotics—namely, *Lactobacillus reuteri, Lactobacillus plantarum, Lactobacillus acidophilus, Lactobacillus rhamnosus* and *Bifidobacterium longum*—have been utilized in clinical trials and have significantly improved autistic and GI outcomes. These periods of trial ranged from three weeks to six months [170,173,174,175]. In a randomized control trial, which was carried out on 75 infants, where *Lactobacillus rhamnosus* was administered in 40 infants and a placebo in the remaining infants in control group for the first six months, the outcome revealed that the early administration of probiotic may potentially reduce the risk of developing ASD. These infants were followed over 13 years and 6 out 35 infants in the control group were diagnosed with either Asperger’s syndrome (now part of ASD) or attention deficit hyperactivity disorder (ADHD), while none were diagnosed in the probiotic group [175]. It is also worth mentioning that the a causal link between maternal diet, altered gut microbiome and social behaviour was demonstrated in a breakthrough study where the administration of *Lactobacillus reuteri* over four weeks in rodent offspring born to mothers fed with a high-fat diet with reduced gut microbial diversity, particularly *Lactobacillus reuteri*, was able to significantly improve social behaviour [163]. However, one of the recent systematic reviews has pinpointed the lack of evidence in supporting the beneficial roles of probiotics and prebiotics in ASD and ascertained that more studies are needed to validate the benefits of these interventions in ASD subjects [176].

In terms of antibiotics, vancomycin and metronidazole have been used in treating ASD symptoms. However, metronidazole is not a preferred choice due to its possible risk of causing systemic adverse effects [177]. There is one case report on the administration of amoxicillin over a 10-day course which deemed to improve autism symptoms in a child as reported by his parents [178]. However, in rodent models, the maternal use of oral antibiotics (non-absorbable sulfonamide, neomycin, bacitracin, pimaricin) either during preconception or early gestation period yielded offspring with anxiety-like behaviours and impaired social interactions [179,180]. Further analysis of the faecal samples of the offspring exposed to antibiotics also showed a 50% reduction in the abundance of the *Lactobacillales* and increased *Clostridium*, thus implying that early exposure of antibiotics have a negative impact on the behavioural outcome in offspring [179].

### 6.3. Fecal Microbial Transplant (FMT) and Microbiota Transfer Therapy (MTT)

In a study using mice models, it was demonstrated that the colonization of germ-free mothers with microbiota obtained from SPF mice 30 days prior to mating was able to “normalize” the behavioural outcome in the germ-free mice while the same produced no changes in adult germ-free mice. The elevated stress hormones level was also reversed through colonization of the germ-free mice with the microbiota of SPF mice before 6 weeks of age [124]. This does not only indicate the importance of maternal microbiome at the time of conception but also the need for an earlier intervention for an improved behavioural outcome, which may be challenging in human subjects as the ASD-like behaviour may not be apparent during the perinatal period. In an open-label study involving 18 children diagnosed with ASD, the MTT involving antibiotic treatment for an initial two weeks, followed by extended faecal microbiota transplant (FMT) over a period of 7–8 weeks, significantly improved GI disturbances (constipation, diarrhoea, abdominal pain, indigestion) as well as ASD’s behavioural symptoms. Further, improved bacterial diversity with significant increase in *Bifidobacterium*, *Desulfovibrio* and *Prevotella* were observed and all these changes persisted for eight weeks after the cessation of treatment [138]. Despite its promising benefits, the faecal transplantation may possess the risk of transmitting norovirus and some autoimmune conditions (rheumatoid arthritis, Sjogren’s syndrome), aspiration and even inducing obesity in recipients [181,182]. However, these are theoretical speculations which should not be simply disregarded.

## 7. Discussion

While the relation between microbiome and ASD is extensive, the immense possibility of the microbial role in ASD is indisputable, inclusive of whether viewed from an aetiological point of view or its association with various comorbidities affecting the ASD population. However, research efforts in understanding the exact mechanism involved in the context of ASD are still largely inadequate; relationships are yet to be established as causal and are rather associative at this stage, mainly due to the heterogeneous nature of this disorder. Although germ-free and animal models have been utilized to a great extent to demonstrate the clear associations of microbes in host physiology, coupled with the fact that human genome resembles 85% of mouse genome, it is near impossible to create germ-free human samples and eliminate all the confounding factors in establishing the same outcome [183]. The genetic makeup, age, sex, life exposure, environmental influences, medication and comorbidities are all possible confounding factors which need to be carefully defined in human samples [16]. Further, a larger sample size is required to affirm the clinical outcomes especially those involving microbiota-based interventions such as dietary, probiotics and faecal experiments. The influence of geographical locations and diet in ASD and its associated microbiota profile are becoming more relevant; therefore, studies fostering larger cohorts from diverse geographical locations with standardized specifications (e.g., age, gender, comorbidities) are needed to understand if the findings are consistent across the different locations and dietary habits.

Clinically, ASD patients are managed by a multi-disciplinary healthcare team primarily by a child psychiatrist, paediatrician, clinical psychologists and other physicians as per the associated comorbidities, although a regular follow-up and monitoring remains largely questionable. The role of parents and caregivers are of utmost importance in ASD. Although many maternal factors and few paternal factors have been associated with the risk of ASD, a proper parental counselling should be provided to ensure a rational understanding and awareness about ASD development. A prenatal counselling to avoid potentially teratogenic factors especially in mothers with underlying medical or psychiatric comorbidities and those with existing ASD children should not be neglected. However, it has be acknowledged that the maternal influence has been mainly described using animal models and no defined relationship between maternal factors and ASD occurrence have been established. This gap in the existing studies need to be understood to ensure the proper channelling of information to the parents, for this may possibly trigger a detrimental emotional response (anxiety, paranoia), especially in mothers who are either planning to conceive or have given birth to children with ASD. The knowledge about the availability of genetic testing should also be given to parents who wish to conceive or those with a known familial risk of neurodevelopmental disorders.

The incorporation of microbial knowledge in clinical context of ASD management provides a promising insight into defining the neurodevelopmental disorder using potential clinical biomarkers, although no definite classification of biomarkers have been established and widely adapted to date [40,75]. Clinical trials revolving around the microbial field are inadequate with a smaller sample size, lack of large population-based studies, and this is certainly attributable to the highly heterogenous and complex nature of ASD itself. The potential clinical intervention targeting gut microbiome which include prebiotics, probiotics, antibiotics, dietary and supplementary interventions, faecal microbial transplant (FMT) and a modified protocol of FMT, namely microbiota transfer therapy (MTT), are important breakthrough interventions which need to be carefully evaluated and validated using double-blinded, randomized, controlled trials involving larger sample size and standardized treatment regimens and durations. FMT and MTT seem the most promising treatments in restoring gut dysbiosis in ASD, but the safety and tolerability in a long run is still questionable [138]. The various clinical interventions have to be carefully evaluated and catered based on individual suitability and requirements. In the management of highly heterogenous disorder like ASD, personalized treatment based on appropriate clinical judgement will be more beneficial.

Although ASD has been largely identified as a childhood disorder, it should not be forgotten that the adult population is not spared from the gigantic wave of ASD. Epidemiological studies on adult autism is very limited and still remains a poorly explored topic. A survey on psychiatric morbidity in adults carried out in England revealed the prevalence rate of autism is about 1% in adults, affecting more males than females, consistent with the findings involving the cohort of children [184]. Adults with autism has also been reported to be at greater risk of socially deprived and isolated, lack of financial security and poor access to specialist care other than having poor physical and mental health outcomes [185]. The children with ASD who will eventually grow into adults should be examined and followed-up closely and consistently.

## 8. Conclusions

ASD has a major health and economic burden to the affected population and care providers. It is becoming an alarming global epidemic which calls for a greater attention and effort. The wide range of serious health comorbidities associated with ASD is often a huge concern when it comes to clinical interventions. The possible explanation and relation of microbes in terms of aetiology and ASD comorbidities point to the need for an integrative approach in treating ASD subjects. Newly emerging fields of research, such as epigenetics, provide more integrative insights and approaches in directing future research, incorporating knowledge of the microbiome. The study of epigenetics incorporating the microbiome reflects the highly dynamic process which is involved in shaping and reprogramming the growth and development of an individual. The systematic identification of environmental factors which interfere with gene regulation could possibly create new avenues in the clinical management of ASD. The treatment approach in ASD should be more individualized to ensure the best clinical outcomes. Diet and probiotics are important and promising breakthrough microbiota-based interventions in the context of ASD. These interventions have better feasibility and an easier method of administration with less known side effects compared to other interventions. Research efforts should be expanded and focused in this direction to ascertain the clinical efficacy of these interventions.

## Glossary

Microbiome	Collectively refers to the genetic material of the entire microbial community residing on and in human body encompassing bacteria, eukaryotic viruses, fungi, protozoa, archaea and bacteriophage.
Microbiota	Refers to the entire microbial community residing on and in human body encompassing bacteria, eukaryotic viruses, fungi, protozoa, archaea and bacteriophage.
